# Formation of Two-dimensional Electron Gas at Amorphous/Crystalline Oxide Interfaces

**DOI:** 10.1038/s41598-017-18746-4

**Published:** 2018-01-10

**Authors:** ChengJian Li, YanPeng Hong, HongXia Xue, XinXin Wang, Yongchun Li, Kejian Liu, Weimin Jiang, Mingrui Liu, Lin He, RuiFen Dou, ChangMin Xiong, JiaCai Nie

**Affiliations:** Department of Physics, Beijing Normal University, Beijing, 100875 China

## Abstract

Experimentally, we found the percentage of low valence cations, the ionization energy of cations in film, and the band gap of substrates to be decisive for the formation of two-dimensional electron gas at the interface of amorphous/crystalline oxide (a-2DEG). Considering these findings, we inferred that the charge transfer from the film to the interface should be the main mechanism of a-2DEG formation. This charge transfer is induced by oxygen defects in film and can be eliminated by the electron-absorbing process of cations in the film. Based on this, we propose a simple dipole model that successfully explains the origin of a-2DEG, our experimental findings, and some important properties of a-2DEG.

## Introduction

Two-dimensional electron gas (2DEG) formed at an oxide interface is a valuable system for exploring the physics of superconductivity^1–3^, magnetism^4–6^, and their coexistence^7–9^. The origin of 2DEG is also a very important topic warranting thorough research. 2DEG systems have two main types: one type is c-2DEG, it forms at a crystalline/crystalline interface (c-interface), such as crystalline-LaAlO_3_/SrTiO_3_ (c-LAO/STO)^10^; the other type is a-2DEG, it forms at an amorphous/crystalline interface (a-interface), such as amorphous-LaAlO_3_/SrTiO_3_ (a-LAO/STO)^11^. They have similar physical properties in terms of superconductivity^1,12^, potential-well depth^13^, and so on. a-2DEG also has many notable differences compared with c-2DEG, such as less lattice strain at the interface, a higher interface carrier density (*n*_*s*_), and considerably fewer requirements for growth. For example, a high growth temperature and TiO_2_-terminated substrates are not needed for STO-based a-2DEG^14–16^. Unlike c-2DEG, the critical thickness of a-2DEG formation (*t*_*c*_) and the electronic transport properties depend highly on the growth conditions, especially the oxygen pressure^14,15^. These differences make a-2DEG a powerful, controllable, and easily manufactured 2DEG system. However, research on a-2DEG is relatively scarce, and its origin requires in-depth exploration.

The a-interfaces are quite different from the c-interfaces. Previous researches have found that the properties of films and the polar discontinuity at the interfaces are the key factors in c-interface systems^17–19^. However, the properties of amorphous films are quite different from crystalline films and there is no polar discontinuity at a-interfaces. Besides, the much lower growth temperature, the amorphous films and so on make the physics processes happened at a-interfaces to be very different from c-interfaces, such as the role of oxygen vacancies^14,15^. So that, the experimental and theoretical findings in c-interfaces system cannot be directly applied to a-interfaces.

Current studies conjecture a-2DEG originates from oxygen vacancies that form in the substrate near the interface (oxygen vacancy theory)^20–22^. In this theory, oxygen ions diffuse from the STO substrate to the film, causing high-concentration oxygen vacancies to form at the STO side near the interface, thus generating a-2DEG. This theory is important and innovative. During experiments, we found that oxygen vacancies are indeed a critical factor; however, other fundamental mechanisms may be involved in the formation of a-2DEG.

## Results

### The influence of films on a-2DEG

**Figure 1:**
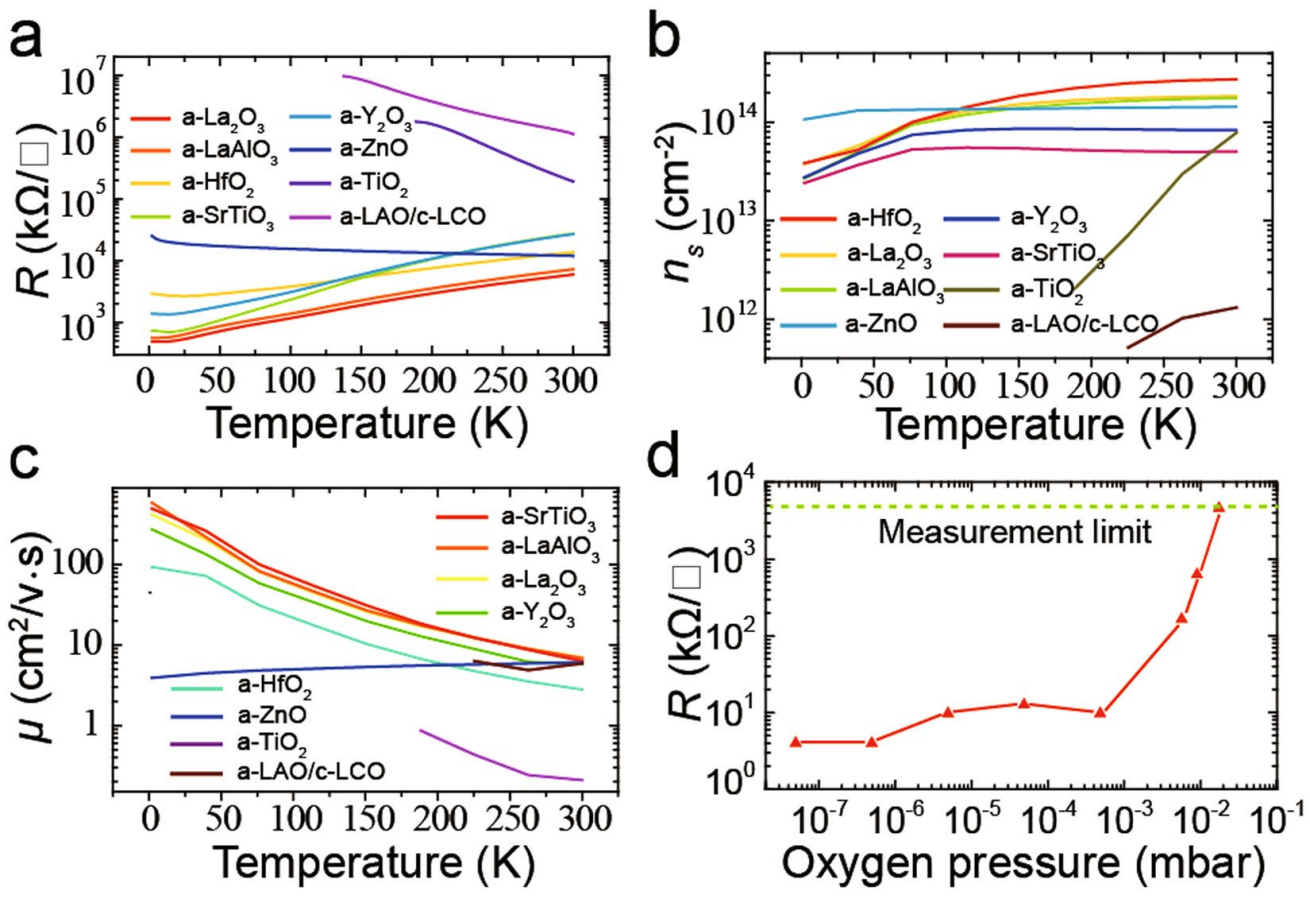
The electrical transport properties of different heterojunctions. (**a**–**c**) Temperature-dependence of interfacial resistance (**a**), *n*_*s*_, (**b**) and mobility (**c**) for different STO-based interfaces. Because SrO reacts rapidly with vapor and CO_2_ in the air, the electrical transport properties of the a-SrO/STO interface were not measured. (**d**) The resistance of LAO/STO interfaces for LAO films grown under different oxygen pressures.

To explore the origin of a-2DEG, we grew a-LaAlO_3_/c-LaCrO_3_/STO (a-LAO/c-LCO/STO) heterojunctions. 2DEG cannot form at a-LCO/STO and c-LCO/STO interfaces^23^; according to the oxygen vacancy theory, the oxygen ion diffusion from c-LCO to STO can be ignored. Thus, the c-LCO layer can act as an effective isolation layer to prevent O^2-^ ion diffusion. c-LCO film was first grown through pulsed laser deposition (PLD) on an STO substrate with the oxygen pressure at 1 × 10^−2^ mbar, temperature at 700 °C, and laser fluence at 0.8 J/cm^2^. The interfaces and c-LCO films were all insulating. Next, a-LAO film was grown under very low oxygen pressure (3 × 10^−8^ mbar) at room temperature. We found that a-2DEG can form even when the c-LCO thickness is approximately 5 nm (Fig. 1(a)), but when the c-LCO layer is thicker, the a-2DEG disappears. And the interfacial mobility (Fig. 1(c)) is similar with the a-LAO/STO interface which has a 2DEG located on STO side^13,24^. These indicate that the a-2DEG is located on STO side, not in the c-LCO layer. However, there is a contradiction between oxygen vacancy theory and experiments. If a-2DEG results from the oxygen vacancies at the STO side, a 5-nm c-LCO film is sufficiently thick to prevent the formation of oxygen vacancies and a-2DEG in the substrate, because c-LCO is an effective isolation layer that is thicker than the oxygen vacancy distribution depth in STO (approximately 2 nm^22,25^). Furthermore, our experiments also showed that the main origin of a-2DEG should be the physical process occurring in the film, not in the substrate or interface. The growth oxygen pressure can influence the formation of a-2DEG (Fig. 1(d)), a-2DEG cannot form if the amorphous films were grown in a high oxygen pressure^14,15^. As we know the lower growth oxygen pressure can induce a higher oxygen defects density of oxide films (*n*_*ox*_). The defining influence of growth oxygen pressure means that the *n*_*ox*_ of films maybe the key factor in the formation of a-2DEG.

For an in-depth investigation, we grew various amorphous oxide films on STO substrates through PLD under low oxygen pressure (4 × 10^−8^ mbar) at room temperature, the laser fluence is 0.8 J/cm2 and the frequency is 1 Hz. a-2DEG formed at the interfaces for amorphous SrO, ZnO, HfO_2_, TiO_2_, La_2_O_3_, Y_2_O_3_, Al_2_O_3_, LaAlO_3_, and SrTiO_3_ films, but not for MnO_2_, Cr_2_O_3_, Fe_2_O_3_, NiO, CeO_2_, LaMnO_3_, LaCoO_3_, and LaCrO_3_ films (Fig. 1(a)). The semiconductor behavior of the a-ZnO/STO interface is caused by a decrease in mobility while its interface carrier density (*n*_*s*_) is nearly constant (Fig. 1(b,c)). This means that the a-2DEG at an a-ZnO/STO interface is as stable as other metallic interfaces. By contrast, the a-2DEG of a-TiO_2_/STO is unstable due to its rapidly decreasing *n*_*s*_ with a reduction in temperature (Fig. 1(b,c)). It is worthy to notice that SrO and La_2_O_3_ can be reacted by H_2_O and CO_2_ in air, SrCO_3_ and La(OH)_3_ will form in films. In fact, we found that the interfacial conductivity of a-SrO/STO and a-La_2_O_3_/STO is unstable when exposed to the air, especially for a-SrO/STO interfaces. If a-2DEG results from the oxygen vacancies at the STO side, the chemical reaction of films may not have so obvious effect on the interfacial conductivity.

Oxygen vacancy theory suggests that a-2DEG can form when the heat of metal oxide formation per mole of oxygen is lower than −250 kJ/(mol O) and the work function of metal is in the range of 3.75–5.00 eV^21^. With this, a-2DEG should not form when grown on SrO, La_2_O_3_, and Y_2_O_3_ because of their work function (2.59 eV for Sr, 3.5 eV for La, and 3.1 eV for Y). Moreover, a-2DEG should form when grown on Cr_2_O_3_ or LaCrO_3_, and a-TiO_2_/STO interface should be metallic^26^. These contradict the experimental results.

### The valence and ionization energy of cations

**Figure 2:**
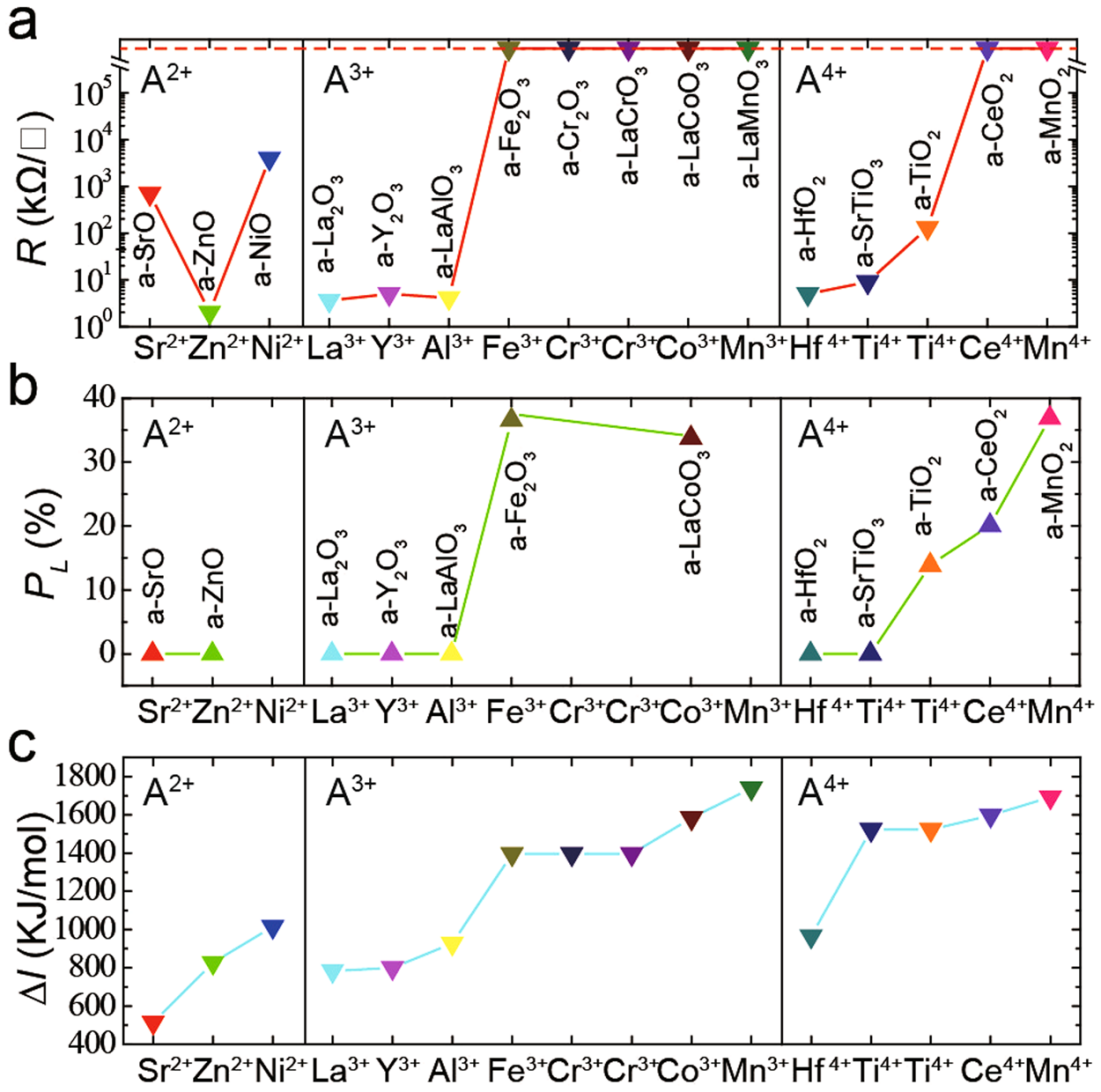
The relationship between *R*, *P*_*L*_ and Δ*I*. The interfacial resistance (**a**), *P*_*L*_ (**b**) and Δ*I* ^34^ (**c**) for various cation types. The shown *R* and *P*_*L*_ are the experimental data of different amorphous-oxide/STO heterojunctions, the corresponding amorphous-oxide films are shown in the vertical texts. The red dashed line in **(a)** is the measurement limit. These cations are divided into three groups (A^2+^, A^3+^ and A^4+^) on the basis of their valence. We set the *P*_*L*_ of Sr^2+^, Zn^2+^, Hf^4+^, La^3+^, Y^3+^, and Al^3+^ as 0, because they generally have only one type of valence. The critical values of Δ*I* differed among the three groups (approximately 1015 kJ/mol for A^2+^ cations, approximately 1395 kJ/mol for A^3+^ cations, and approximately 1597 kJ/mol for A^4+^ cations). The *R* of a-SrO/STO interface should be markedly lower than the shown value because the chemical reaction between SrO and air damaged the interfacial conductivity.

Because amorphous films have no crystalline structure and are grown on the same substrate, the main difference between these interfaces is the cations in films. Therefore, through X-ray photoelectron spectroscopy, we measured the valence of the cations in some of the films (see Fig. S1), which is the most important property of cations. Notably, the percentage of low-valence cations (*P*_*L*_) in the films is closely related to the resistance of a-2DEG (Fig. 2(a,b)). a-2DEG can form only when the effective *P*_*L*_ is lower than approximately 20%. In the present study, we took the highest *P*_*L*_ as the effective *P*_*L*_ for films that had several cation types, because we found that cations with the highest *P*_*L*_ dominate all other cation types. A close relationship also exists between a-2DEG and the ionization energy of cations (Fig. 2(a,c)). a-2DEG can form only if the ionization energy difference (Δ*I*) of both cation types in the film is less than a critical value, which depends on the valence of the cations (Fig. 2(c)). Δ*I* is the difference between the *N*th and (*N*−1)th ionization energy (*I*_N_ and *I*_N−1_) of cations, where *N* is the normal valence of cations (e.g., Δ*I* = *I*_3_ – *I*_2_ for Al^3+^ cations). As with *P*_*L*_, in films with several types of cation, the cations with the highest Δ*I* dominate other cation types. It is worthy to notice that the cations with lower Δ*I* have an influence on the dominant one, for example, the Ti^4+^ in a-STO film have a much lower *P*_*L*_ than the Ti^4+^ in a-TiO_2_ film due to the influence of Sr^2+^ cations (Fig. 2(b)). The semiconductor behavior of a-TiO_2_/STO interfaces can be understood by considering the relatively high *P*_*L*_ and Δ*I* of Ti^4+^ compared with the other cations, which can result in a-2DEG with the same valence as Ti^4+^ (Fig. 2). All of our findings indicate the critical role of the type of film used in a-2DEG formation.

### The influence of the band gap of substrates

**Figure 3:**
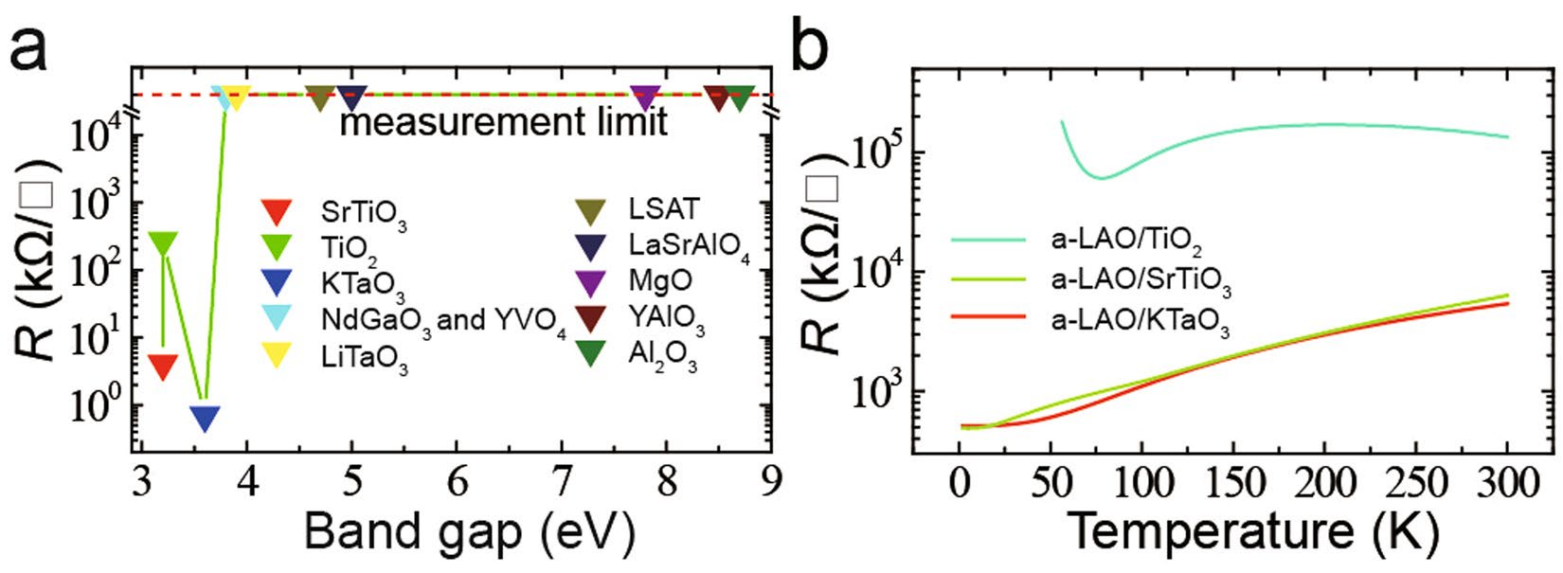
The influence of substrates. (**a**) Relationship between the interface resistance and band gap of the substrate^35–45^ for a-LAO films grown on various substrates. (**b**) Temperature dependence of the interface resistance for a-LAO films grown on TiO_2_, SrTiO_3_, and KTaO_3_ substrates.

To explore the influence of the substrate on the formation of a-2DEG, we grew a-LAO on different crystalline substrates under a low oxygen pressure (4 × 10^−8^ mbar), the laser fluence is 0.8 J/cm2 and the frequency is 1 Hz. a-2DEG only formed on SrTiO_3_, KTaO_3_, and TiO_2_ (rutile) substrates (Fig. 3(a)). The different behaviors of a-LAO/SrTiO_3_, a-LAO/KTaO_3_, and a-LAO/TiO_2_ interfaces (Fig. 3(b)) indicate the marked influence the substrates have on the properties of a-2DEG as the a-2DEG localized in the substrates. Notably, we found that the a-2DEG formation and substrate band gap were closely related. A substrate band gap of less than or equal to approximately 3.6 eV is necessary for a-2DEG formation (Fig. 3(a)). This result cannot be explained by the oxygen vacancy theory. The bare substrates are all insulating and the conduction bands of them are all empty of electrons. The metallic behaviors of some interfaces means the conduction band of these substrate should not be empty when a-LAO was grown on them. So that, there must exist a charge transfer process injecting electrons into the conduction band. The band gap should be the potential barrier that this process has to overcome. If the band gap is too large to overcome, no electrons can be injected into the condition band of substrates and a-2DEG cannot form.

## Discussions

### The charge transfer process

Given the high *n*_*ox*_ induced by the low growth oxygen pressure, a large number of free electrons must be present in film to maintain the electric neutrality. The high *P*_*L*_ means that all or most of these electrons are absorbed by cations in the film and cannot participate in other physical processes. Considering the relationship between *P*_*L*_ and a-2DEG, we conjectured that these electrons should be the source of a-2DEG and that there should be a charge transfer process causing these electrons to transfer to the interface. The high *n*_*ox*_ is a key factor. Another important factor is the electron-absorbing process of the cations, because it can disrupt the a-2DEG formation process and result in low-valence cations. We conjectured that Δ*I* indicates the electron-absorbing ability of the cations, with a larger Δ*I* indicating stronger electron-absorbing ability and higher *P*_*L*_. For all cation types in the film, Δ*I* being smaller than the critical value is a critical requirement for forming a-2DEG.

Current theories offer a poor explanation for *t*_*c*_. Some researchers believe that *t*_*c*_ is the film thickness to form a conducting channel in STO^22,25^, while others argue that it is the film thickness that prevents the interface from being reoxidized by air^27^. However, we have found that the oxygen vacancy is not the main origin of a-2DEG, and these explanations should not be correct. The band gap limit of substrates and the influence of *P*_*L*_ on a-2DEG formation suggest that a charge transfer process from film to interface may exist. The charge transfer from the film to the interface is the origin of c-2DEG and *t*_*c*_ is also an important property for c-2DEG^28^. For c-interface, the energy of valence band of film will increase with increasing film thickness due to the polar discontinuity at interface. *t*_*c*_ is the film thickness that the energy of valence band of film is equal to the conduction band of substrate. The c-2DEG system is an great inspiration for a-2DEG case because of the similar properties of them. Considering the above mentioned, we believe that the formation of a-2DEG may also be a charge transfer process which can inject electrons into the conduction band of substrate. Thus, the semiconductor behavior of a-LAO/c-LCO/STO (Fig. 1(a)) should result from the c-LCO layer, which weakens the charge transfer from the film to the substrate. On the basis of the experimental findings and the origin of c-2DEG^28^, we conjectured that the electrical potential energy in the film should be the cause of charge transfer. Unlike c-2DEG, however, the substrates have a maximum band gap (3.6 eV) to form 2DEG (Fig. 3(a)), and the variable *t*_*c*_ depends strongly on the growth oxygen pressure^14,15^. Thus, we believe that the electrical potential energy should be caused by the high *n*_*ox*_ of the film, and it does not diverge with an increase in film thickness. Here, we propose a simple dipole model based on the high *n*_*ox*_ of the film to semiquantitatively explain the origin of a-2DEG.

### The dipole model

**Figure 4:**
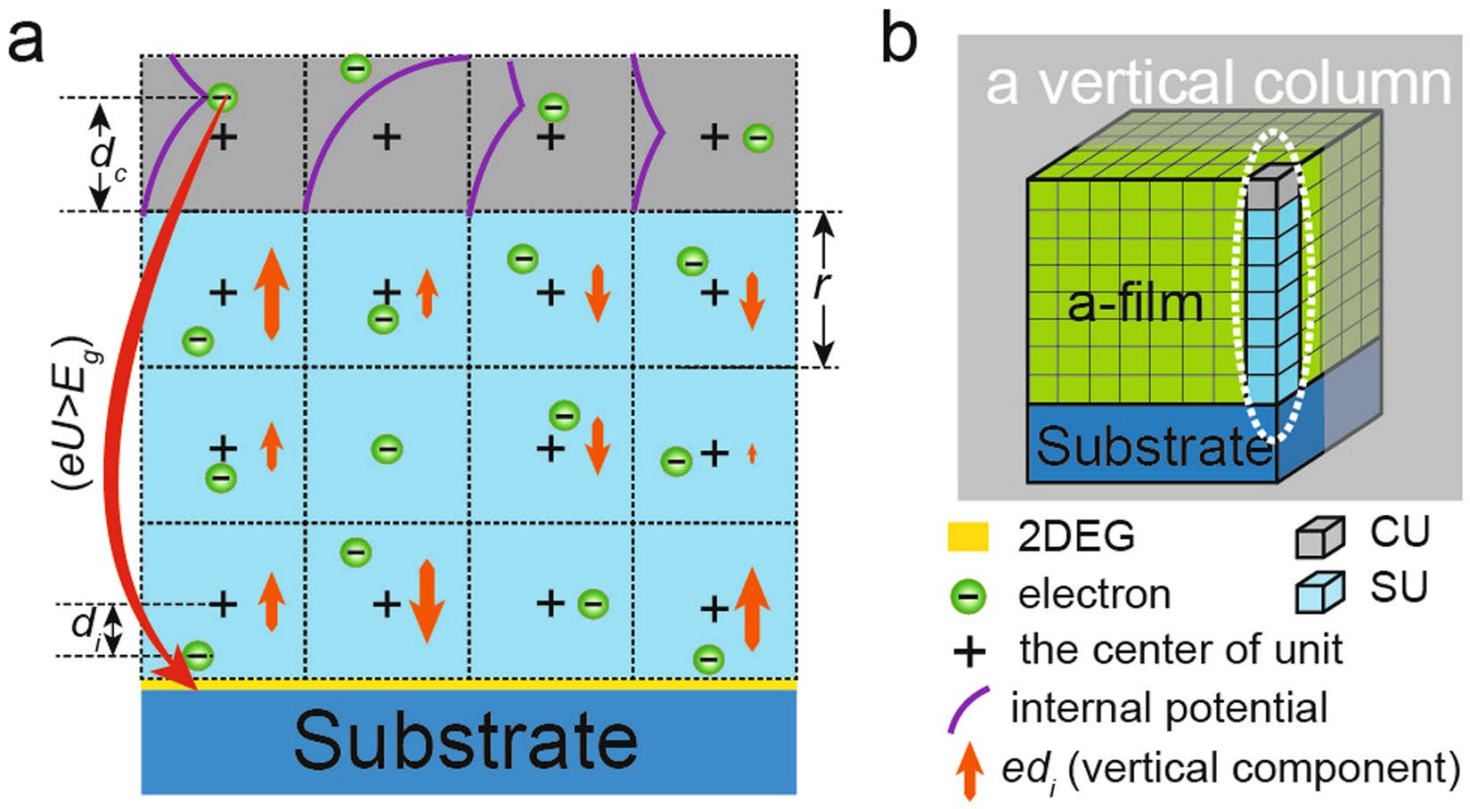
Schematic of the dipole model. (**a**) is the cutaway view of (**b**), the gray plane in (**b**) is the cut plane.

The amorphous film can be regarded as the combination of ions and electrons generated by the high *n*_*ox*_. To simplify our model, we assumed roughly that the electrons were distributed uniformly in the positively charged background, which comprises all ions in the film and is also uniform. Under this assumption, every electron occupies a surrounding volume of (2*n*_*ox*_)^−1^ on average and the total charge of the volume is +*e*. Here, *e* is the charge of one electron. One electron and the surrounding background constitute an electric neutrality unit, and the amorphous film can be regarded as closely stacked units of this type (Fig. 4(b)) when assuming one unit is a cube.

When the electron is not located at the center of the unit, the unit is an electric dipole and influences the potential energy of other electrons. The electron also has an internal potential energy that depends on the location of the electron inside of one unit (Fig. 4(a)). Therefore, the potential energy of one electron (*eU*) consists of two parts, one is generated by the electric dipole moment of other units, and the other is the internal potential. When the total potential of one electron is larger than the band gap of the substrate (*eU* > *E*_*g*_), this electron can transfer to the interface and form a-2DEG. Notably, *eU* is microcosmic and there is no macroscopic electric field in the film. Thus, when the amorphous film is annealed in oxygen, the recovery of oxygen stoichiometry destroys *eU* and a-2DEG, as observed in previous experiments^14,15^. Besides, the chemical reaction of films will obviously change the components of films. It will destroy *eU* and weaken the interfacial conductivity, such as the decreasing interfacial conductivity of a-SrO/STO when exposed to air.

### Model details and results

In the calculation, we divided the units into two types: charge-transfer-units (CUs) and the stable-units (SUs) (Fig. 4(a,b)). The CUs are the top units in a vertical column because the electrons in them can achieve the highest potential and they are the most likely to transfer to the interface. The other units are SUs. To simplify the calculation, we considered only the units in the same vertical column (perpendicular to the surface) (Fig. 4(b)). For this reason, our calculation results deviated slightly from actual situations, especially situations in which the film is thick. In one vertical column, the effective electric dipole moment of one SU is *ed*, which is the vertical component of the electric dipole moment. Here, *d* is the vertical component of the distance between the electron and the center of the unit. The internal potential depends on the location of the electron in the vertical direction (*d*_*c*_). Taking the *n*th unit as CU when a vertical column has *n* units, the total potential *eU* can be written as Eq. (1)

1 $$eU=\sum _{i=1}^{n-1}\frac{e{d}_{i}}{4\pi {{\rm{\varepsilon }}}_{r}{{\rm{\varepsilon }}}_{0}{(n-i)}^{2}{r}^{2}}+\frac{e{n}_{ox}{d}_{c}^{2}}{{{\rm{\varepsilon }}}_{r}{{\rm{\varepsilon }}}_{0}}\,(-\frac{r}{2}\le {d}_{i}\le \frac{r}{2},0\le {d}_{c}\le r)$$

The first item of *eU* is generated by the electric dipole moments of the SUs, the second item is the internal potential energy of the CU. There term *d*_*i*_ is *d* of the *i*th unit, *r* = (2*n*_*ox*_)^−1/3^ is the side length of one unit, and (*n*−*i*)*r* is the distance between CU and the *i*th SU roughly.${{\rm{\varepsilon }}}_{r}\,$is the relative dielectric constant (${{\rm{\varepsilon }}}_{r}$= 13 for a-LAO^29^).

**Figure 5:**
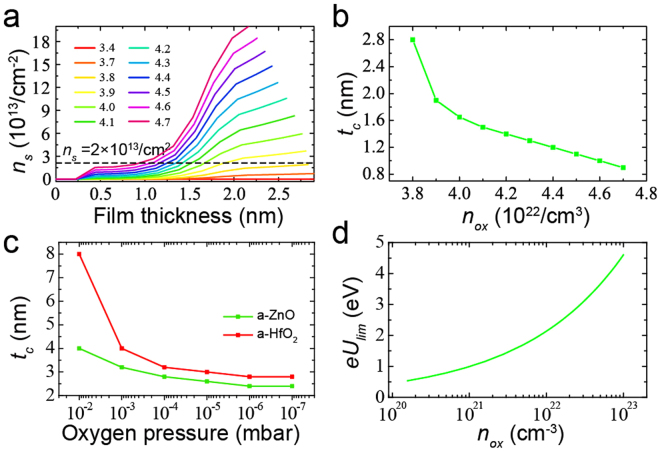
The calculation results. (**a**) The film thickness dependence of calculated *n*_*s*_ for different *n*_*ox*_ (10^22^/cm3). (**b**) The calculated *t*_*c*_ for different *n*_*ox*_ when taking *n*_*s*_ = 2 × 10^22^/cm3 as a criterion (black dashed line in (**a**)). (**c**) The experimental value of *t*_*c*_ of a-ZnO/STO and a-HfO_2_/STO. (**d**) The *eU*_*lim*_ for different *n*_*ox*_.

Because of the random distribution of electrons, *d*_*c*_ and *d*_*i*_ are random and the *eU* differs for different electrons in the film. *eU* can be positive or negative, so that only part of the electrons can transfer to the interface. The percentage of transferred electrons (*eU* > *E*_*g*_) is *p*, the interface carrier density *n*_*s*_ = *p*(2*n*_*ox*_)^2/3^, where (2*n*_*ox*_)^2/3^ is the sheet electrons’ density in the CU layer. Calculating *p* is the key to obtaining *n*_*s*_. We calculated the *n*_*s*_-thickness cures of a-LAO/STO for different *n*_*ox*_ with a computer program (Fig. 5(a)). We find that when *n*_*ox*_ is smaller than a certain value (3.4 × 10^22^/cm^3^), no charge transfer can occur and no a-2DEG can form at the a-LAO/STO interface. Figure 5(a) also show that the *n*_*s*_ will gradually increase with increasing film thickness when the film thickness is near *t*_*c*_. And the *n*_*s*_ may reach a stable value when the film is thick.

2 $$e{U}_{lim}=\frac{e{n}_{ox}^{1/3}}{{2}^{2/3}{{\rm{\varepsilon }}}_{r}{{\rm{\varepsilon }}}_{0}}+\frac{e\pi {(2{n}_{ox})}^{1/3}}{48{{\rm{\varepsilon }}}_{r}{{\rm{\varepsilon }}}_{0}}$$

**Figure 6:**
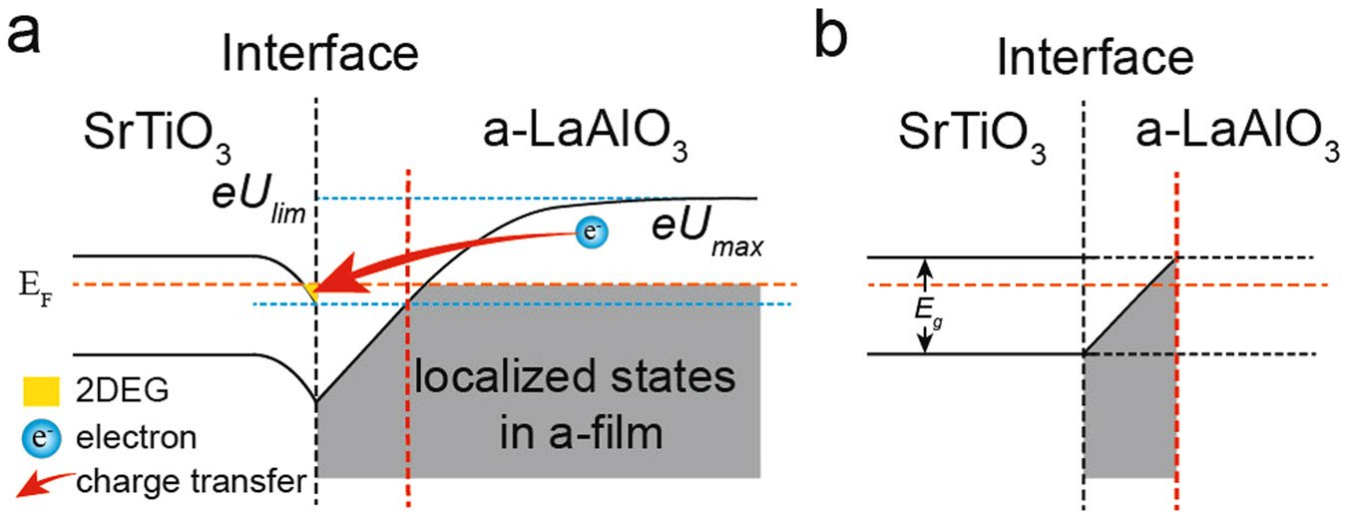
Band alignment of a-LAO/STO. Schematic of the band alignment of the a-LAO/STO interface when the film is thicker (**a**) or thinner (**b**) than the critical thickness of the charge transfer (red vertical dashed lines).

We found that *eU* has a maximum value (*eU*_*max*_, Fig. 6) versus *n* for a certain *n*_*ox*_ when set *d*_*i*_ = *r*/2 and *d*_*c*_ = *r*. *eU*_*max*_ becomes enhanced with an increase in *n* (equivalent to an increase in film thickness). Notably, *eU*_*max*_ also has a limit to its value (*eU*_*lim*_, Eq. (2), Fig. 6), which is proportional to *n*_*ox*_^1/3^ when *n* = +∞ (Fig. 5(d)). *eU*_*lim*_ is the maximum potential energy of electrons in the film for a certain *n*_*ox*_ and it is also the origin of the band gap limit for a-2DEG formation (Fig. 3(a)). Thus, we can plot a schematic of the band diagrams of a c-LAO/STO (Fig. 6); with this, the formation of a-2DEG can be clearly understood. The oxygen defects in film will generate a microcosmic electric potential and increase the energy of part of electrons in film with increasing the film thickness. When the energy of those electrons is higher than the conduction band of substrate, a charge transfer from film to substrate will happen and a-2DEG form at the interface. Because a minimum value of *n*_*s*_ is needed to from a macroscopic conducting channel due to the in-gap states in STO^30–33^, the critical thickness for charge transfer (the film thickness when *eU*_*max*_ > *E*_*g*_) is not *t*_*c*_, the critical thickness for a-2DEG formation. Here, we took the film thickness with an *n*_*s*_ that is equal to 2 × 10^13^/cm2 as *t*_*c*_. A *n*_*ox*_-dependent *t*_*c*_ was found (Fig. 5(b)), and this is in agreement with experimental findings (Fig. 5(c)).

**Figure 7:**
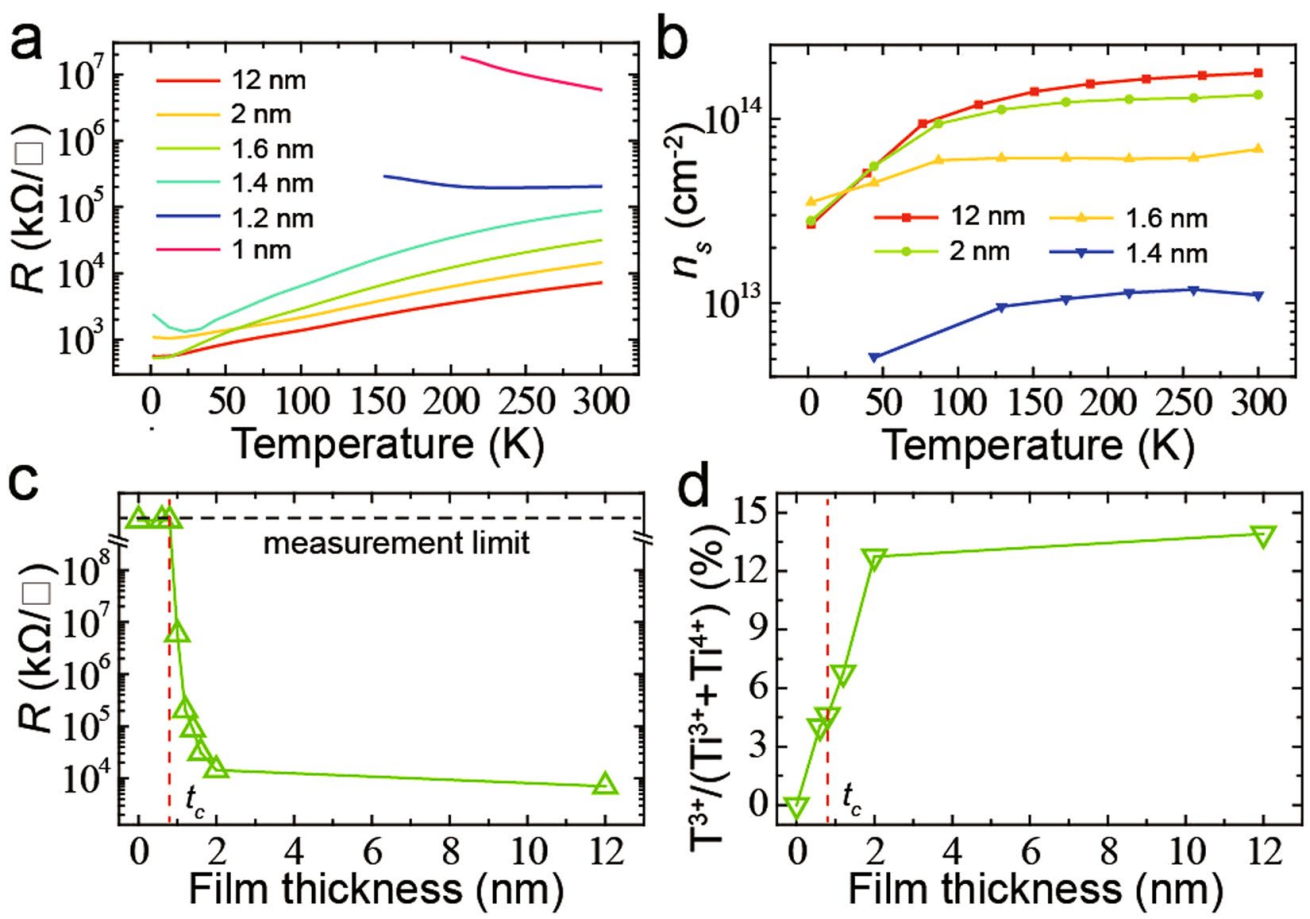
Properties of a-LAO/STO with different film thickness. **(a**–**c)** Temperature-dependence of interfacial resistance **(a)** and *n*_*s*_ **(b)** for a-LAO/STO interfaces with different film thickness. **(c)** The film thickness dependence of interfacial resistance. **(d)** The film thickness dependence of the percentage of Ti^3+^ cations. The red dashed lines in (**c**) and (**d**) indicate *t*_*c*_.

For an in-depth investigation, we grew a-LAO films with different thickness on STO substrates in an oxygen pressure of 4 × 10^−8^ mbar, at room temperature. The transport properties and the percentage of Ti^3+^ cations on STO side (see XPS data in Fig. S2) was measured (Fig. 7). The *t*_*c*_ of a-2DEG formation is about 0.8 nm (Fig. 7(c)). We found that the a-LAO/STO interfaces show a gradual insulator-metal transition when increasing a-LAO thickness (Fig. 7 (a,c)). The *n*_*s*_ and the percentage of Ti^3+^ cations also gradually increase with increasing a-LAO thickness, and they will reach a stable value when the films are very thick (Fig. 7 (b,d)). These findings are in agreement with our model as discussed above (Fig. 5(a)). We found that Ti^3+^ cations can form at STO side when the interfaces are insulating. That means the critical thickness for charge transfer is smaller than *t*_*c*_ as we suggested. The transferred electrons are mainly trapped by the in-gap states in STO^30–33^ when the film thickness below *t*_*c*_. When increasing the a-LAO film thickness, more electrons will transfer to STO side and result in an insulator-metal transition (Fig. 7(a,c)). All of these findings have shown that our model can match the experimental results very well.

## Conclusions

We grew various amorphous oxide films on STO substrate and grew a-LAO films on different substrates. We found *P*_*L*_, Δ*I*, and the substrate band gap are closely related to a-2DEG formation. These findings indicate that the high *n*_*ox*_ and electron-absorbing ability of cations in film are the key factors of a-2DEG formation. Considering the origin of c-2DEG and the critical thickness of a-2DEG formation, there should exist a charge transfer process caused by the high *n*_*ox*_ of the film. We propose the dipole model to semiquantitatively explain the origin of a-2DEG. The results of our model are in good agreement with our experiments.

## Methods

### The electric properties Measurements

The electric properties was measured using a Physical Properties Measurement System (PPMS). Hall bar structures were prepared on the samples though photoetching. Ultrasonic Al wire bonding was used to connect the interface. The interfacial carrier density and mobility for different interfaces was characterized by measuring the longitudinal resistivity and Hall resistivity.

### The X-ray photoelectron spectroscopy Measurements

The XPS data were obtained using an ESCA Lab250 electron spectrometer (Thermo Scientific Corporation). The x-ray source is monochromatic 150 W Al *Kα* (*hv = *1486.6 eV) radiation. The ultimate energy resolution is 0.45 eV and the measurements were done at room temperature. We measured the core level spectra of different elements of films and used the peak area of different valence cations to calculate the *P*_*L*_. A Shirley background subtraction was used. Because all films are very thick, the substrates have no influence on the measurements.

## Electronic supplementary material

Supplementary Information
