# Supplementary material for: Formation of Two-dimensional Electron Gas at Amorphous/Crystalline Oxide Interfaces
Source: Sci Rep. 2018 Jan 10;8:404. doi: 10.1038/s41598-017-18746-4 (PMC5762893; doi:10.1038/s41598-017-18746-4)
Supplement: Supplementary file 1 — Supplementary Information [file 41598_2017_18746_MOESM1_ESM.pdf]

Supplementary Information:

Formation of Two-dimensional Electron Gas at Amorphous/Crystalline Oxide Interfaces

ChengJian Li, YanPeng Hong, HongXia Xue, XinXin Wang, Yongchun Li, Kejian Liu, Weimin Jiang, Mingrui Liu , Lin He, RuiFen Dou, ChangMin Xiong, JiaCai Nie*

Department of Physics, Beijing Normal University, Beijing, 100875, China

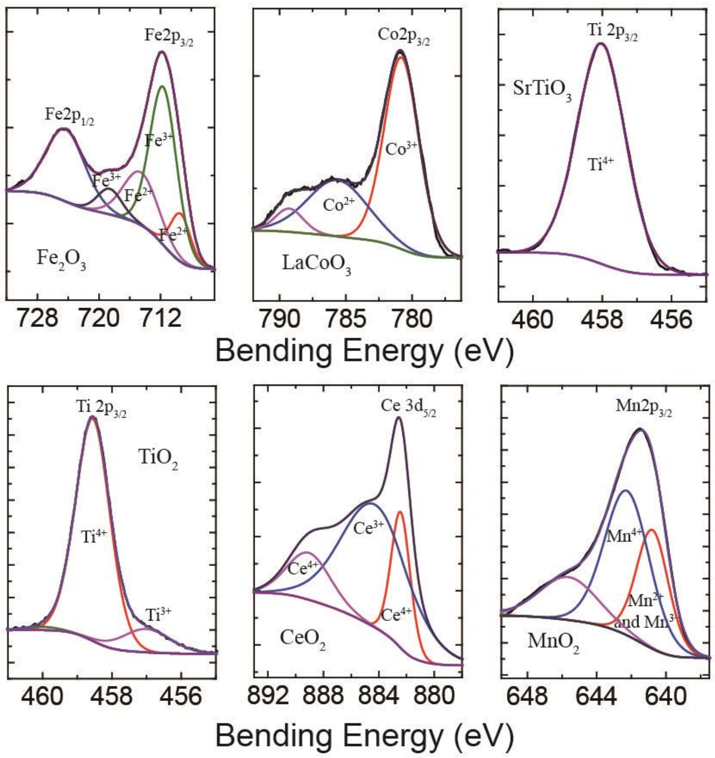

FigS.1 The core level spectra of Fe $2p_{3/2}$, Co $2p_{3/2}$, Mn $2p_{3/2}$, Ti $2p_{3/2}$, Ce$2d_{5/2}$ of different oxide films. We fitted the spectra and used the peak area to calculate the P_L. Shirley background subtraction has been used for all the spectra.

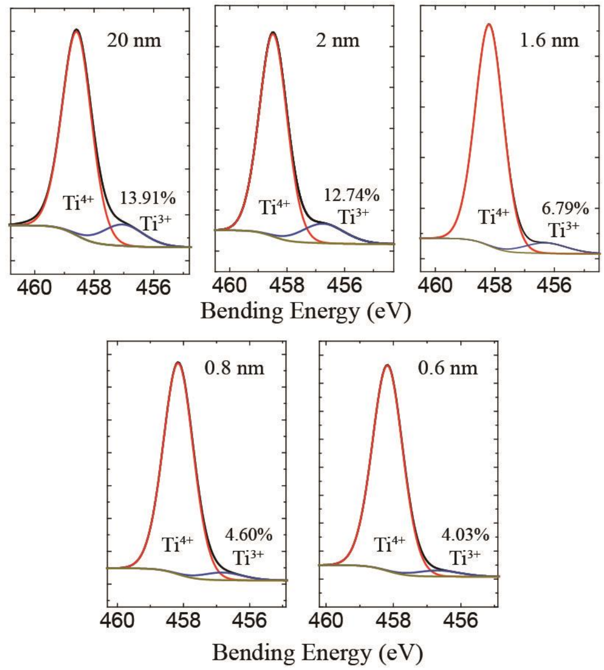

FigS.2 The core level spectra of Ti $2p_{3/2}$ of a-LAO/STO with different films thickness. We fitted the spectra and used the peak area to calculate the percentage of Ti^{3+} cations. Shirley background subtraction has been used for all the spectra. We found that the percentage of Ti^{3+} cations increase with increasing film thickness.
